# Addendum: Stunning of neutrophils accounts for the anti-inflammatory effects of clodronate liposomes

**DOI:** 10.1084/jem.2022052506022023a

**Published:** 2023-06-09

**Authors:** Stephan Culemann, Katharina Knab, Maximilien Euler, Anja Wegner, Hilal Garibagaoglu, Jochen Ackermann, Kim Fischer, Deborah Kienhöfer, Georgiana Crainiciuc, Jonas Hahn, Anika Grüneboom, Falk Nimmerjahn, Stefan Uderhardt, Andrés Hidalgo, Georg Schett, Markus H. Hoffmann, Gerhard Krönke

Vol. 220, No. 6 | https://doi.org/10.1084/jem.20220525 | March 28, 2023

Since publication, it was brought to our attention that the Materials and methods section was incomplete and did not include information on the origin, preparation, and handling of the clodronate liposomes used in our study. We regret this omission and provide the information in this addendum.

The paragraph below has been added to the Materials and methods section. The printed article and PDFs downloaded before June 5, 2023, are missing these details.

## Liposomes

Clodronate liposomes (SKU: C-005) and PBS liposomes (SKU: P-005) were commercially available and purchased from Liposoma BV (https://clodronateliposomes.com, http://clodronateliposomes.org). The concentration of the clodronate in the suspension was 5 mg/ml. Liposome suspensions were injected directly without any further dilutions. For labeling of clodronate liposomes with Vybrant DiD (Vybrant DiD Cell-Labeling Solution, Invitrogen, V22887), 10 µl of the Vybrant dye solution was added to 1 ml clodronate liposome suspension and mixed by gentle shaking. After incubation at 37°C for 20 min (slowly shaking), labeled liposome suspension was centrifuged at 1,500 rpm for 5 min, producing a loose phase of liposomes and an upper aqueous phase. The aqueous phase was removed, and an equivalent volume of PBS was added to the liposomes and mixed well by gentle pipetting. After an additional centrifugation step (1,500 rpm, 5 min) and removal of the upper aqueous phase, an equivalent volume of PBS was added to liposomes to get the original volume.

